# A population‐based study of homelessness, antisocial behaviour and violence victimisation among young adults in Victoria, Australia

**DOI:** 10.1002/ajs4.212

**Published:** 2022-04-13

**Authors:** Jessica A. Heerde, Jennifer A. Bailey, George C. Patton, John W. Toumbourou

**Affiliations:** ^1^ 34361 Department of Paediatrics The University of Melbourne Melbourne Victoria Australia; ^2^ Department of Social Work The University of Melbourne Melbourne Victoria Australia; ^3^ Centre for Adolescent Health Royal Children’s Hospital Melbourne Victoria Australia; ^4^ Murdoch Children’s Research Institute Melbourne Victoria Australia; ^5^ 7284 Social Development Research Group School of Social Work University of Washington Seattle Washington USA; ^6^ 34361 School of Psychology Centre for Social and Early Emotional Development Deakin University Burwood Victoria Australia

**Keywords:** homelessness, longitudinal survey, physical violence, victimisation, young adult

## Abstract

Homeless young adults are at increased risk for contact with the police and are overrepresented in the justice system. This study explored associations between homelessness, antisocial behaviour and violence victimisation using longitudinal panel data gathered through young adulthood. Data were drawn from a state representative population‐based sample of young adults from Victoria, Australia participating in the International Youth Development Study (IYDS; *n* = 2884, 54% female). Participants were surveyed at age 21 years, with follow‐up at ages 23 and 25 years. We examined changes in the prevalence of homelessness and tested hypothesised directional relationships between young adult homelessness, antisocial behaviour and violence victimisation using longitudinal cross‐lagged panel models. Multiple‐group modelling was used to test whether these relationships were moderated by gender. The prevalence of young adult homelessness was highest at age 21 (6.5%), declining at ages 23 (3.9%) and 25 years (2.5%). Results showed that young adult homelessness, antisocial behaviour and victimisation were related cross‐sectionally, but not longitudinally. Gender did not significantly moderate these associations. Findings suggest that the state of homelessness is associated with temporary vulnerability to potentially harmful and problematic situations involving antisocial behaviour and victimisation. These situations are likely to heighten risk for contact with the police and direct physical and psychological harm.

## 1 INTRODUCTION

Homelessness has been defined as having no suitable or permanent occupancy at a residence (e.g., couch surfing) or being unsheltered (e.g., living directly on the streets or in spaces not intended for habitation) or in emergency shelter or temporary accommodation (Australian Institute of Health & Welfare, 2020). Although most Australian young adults experience few health and social problems, those who are homeless face substantial social disadvantage and have uniquely complex health needs, which are often long‐term in nature. Rates of homelessness among Australian young adults continue to be of concern. National prevalence estimates suggest that 41,099 Australian young adults accessed support from specialist homelessness services in 2018–19, representing 14.2% of all clients (Australian Institute of Health & Welfare, 2019). Estimates for the most recent periods of 2019–20 and 2020–21 remain similar (Australian Institute of Health & Welfare, 2020, 2021). Homelessness is associated with higher rates of preventable morbidities and mortality, including those arising from external causes such as violence and victimisation (Aldridge et al., 2018; Heerde & Patton, 2020). Homeless young adults are at increased risk for contact with the police (Alder et al., 1989) and are overrepresented in the justice system (Aldridge et al., 2018). In Australia, 45% of individuals aged 18–24 years entering the justice system experienced homelessness in the preceding month (Australian Institute of Health Welfare, 2019).

Understanding both how the prevalence of homelessness changes across young adulthood, and the extent to which morbidities associated with the state of homelessness are amenable to prevention, is warranted. Three recent studies have drawn attention to the considerable health and social inequities experienced by homeless persons (Aldridge et al., 2018, 2019; Marmot, 2018), including those arising from assault and victimisation, and driven by the multiple personal, social, structural, and economic intersecting vulnerabilities underpinning these inequities (Marmot & Allen, 2014; McNeil et al., 2013). Homelessness in young adulthood is a significant driver of social inequality and poor health outcomes, disrupts education, training and employment, and interrupts the attainment of adult developmental milestones that set the foundation for health, social and economic participation into later life (Koegel et al., 1995; Scales et al., 2016; Van den Bree et al., 2009).

Point‐in‐time counts provide critical data on the number of persons exposed to homelessness on any given night (Auerswald & Adams, 2018). However, they do not provide information on how the prevalence of homelessness changes for individuals over time or across key developmental periods, that is, within person patterns. Longitudinal studies offer critical opportunities to understand how the prevalence of homelessness changes through early adulthood; however, few such studies exist (Davies & Wood, 2018; Heerde et al., 2021, 2022; Morton et al., 2018; Tyler & Bersani, 2008; Van den Bree et al., 2009). An important step in understanding how rates of homelessness change over time is to identify key developmental periods amenable to targeted preventive intervention.

Although not a primary focus of the current study, consideration of longitudinal pathways from adolescent to young adult homelessness is necessary. It is challenging to obtain reliable, robust longitudinal data on homelessness as it is difficult to engage and retain participants (Aldridge et al., 2018; Heerde & Patton, 2020). Due to these difficulties, most prior studies have used data collected retrospectively from selective community based samples of homeless participants or those already established as high risk, without a comparison group of non‐homeless participants (Bearsley‐Smith et al., 2008; Chamberlain & Johnson, 2013; Fitzpatrick et al., 2013; Haber & Toro, 2004). The small number of prospective, population‐based longitudinal studies investigating risk for young adult homelessness have identified various adolescent predictors, which are summarized below.

Prior analyses using data from the Australian subsample of the International Youth Development Study (IYDS) showed that higher levels of family conflict at age 13 uniquely increased risk for any young adult homelessness across ages 21–25, with this association mediated by age 15 peer drug use and interactions with antisocial peers (Heerde et al., 2021). Other findings from the combined US‐Australian IYDS sample showed that academic failure and school suspension increased risk for young adult homelessness (Heerde et al., 2020a). In other analyses using this sample, longitudinal path modelling showed that poor family management strategies at age 13 increased risk for homelessness at age 25, with this association partially mediated by peer drug use, school suspension, academic failure and lower neighbourhood attachment at age 15 (Heerde et al., 2022). Similar findings have been reported in US‐based studies analysing data from the National Longitudinal Study of Youth‐97 (NLSY‐97) and the National Longitudinal Study of Adolescent and Adult Health (Add Health). Across these studies, non‐traditional family structure, poorer family relationships and family socio‐economic difficulties, prior runaway/homelessness episodes, low educational attainment and school adjustment problems, victimisation, poor mental health and illicit drug addiction during adolescence predicted young adult homelessness (Shelton et al., 2009; Sznajder‐Murray et al., 2015; Van den Bree et al., 2009; Williams et al., 2019).

For many homeless young adults, engagement in antisocial behaviour and violence victimisation are commonplace (Baron, 2003; Heerde & Palotta‐Chiarolli, 2020; Kipke et al., 1997; Tyler et al., 2018; Tyler & Schmitz, 2018). Prior review work and meta‐analyses provide an insight into the prevalence of antisocial behaviour and violence victimisation among homeless young adults. Rates of assaulting someone (for reasons other than protection) ranged from 10% to 45%, while rates of having been assaulted varied between 23% and 81% (Heerde et al., 2014). Experiences of violence and victimisation among homeless young adults are heterogeneous and are frequently compounded by histories of childhood abuse and family violence (Heerde & Hemphill, 2019; Tyler & Schmitz, 2018). These experiences are further complicated by social expectations and rules within homeless communities that see physical violence and victimisation accepted as normal when it occurs (Baron, 2003; Gaetz, 2004; Heerde & Palotta‐Chiarolli, 2020; Tyler & Schmitz, 2018), despite these being disparaged and prohibited under broader social laws and regulations, and support being provided for those who have been victimised.

In Australia, studies investigating homeless young adults’ engagement in antisocial behaviour and their victimisation experiences are few. These are typically small, retrospective, cross‐sectional (Alder, 1991; Alder et al., 1989) or qualitative (Heerde & Palotta‐Chiarolli, 2020, 2021; Watson, 2017) studies with data collected from purposefully recruited community‐based samples without a comparison group of non‐homeless young adults and/or with limited follow‐up and high attrition. An exception is the Journeys Home project. Analyses using Journeys Home data have examined living and housing challenges and self‐reported heath and behaviour among a selective sample of participants exposed to homelessness or to high levels of housing insecurity, and receiving welfare support (40% of respondents were 15–24 years of age), over approximately two and half years (Scutella et al., 2013). Study findings suggest childhood experiences of homelessness increase risk for non‐high school completion and adult unemployment (Cobb‐Clark & Zhu, 2017; Scutella et al., 2013), while risk for engagement in violence and victimisation were increased among those experiencing housing insecurity (Diette & Ribar, 2018).

The analysis of longitudinal, prospective data from a population‐based sample, as in the current study, permits an examination of rates of homelessness over time, and an investigation of how homelessness, antisocial behaviour, and violence victimisation are related. Findings from this and other longitudinal studies will provide novel and essential evidence to inform both prevention and intervention strategies and guide the efficient allocation of scarce resources for homeless young adults. For example, if antisocial behaviour is associated with later homelessness in young adulthood, then rates of homelessness may be lowered by greater implementation of tested‐effective programming (Blueprints for Healthy Youth Development, 2019) to reduce young adult antisocial behaviour, such as Communities That Care (Hawkins et al., 2014; Toumbourou et al., 2019). Alternatively, if homelessness is associated with later antisocial behaviour and violence victimisation, then programming focussed on reducing the number of young adults who enter homelessness (e.g., population‐level prevention, the non‐entry of young adults into homelessness from care or justice settings), reducing the length of time young adults experience homelessness, and keeping homeless young adults safe through coordinated intervention responses across sectors should be prioritised (Heerde et al., 2020a, 2022).

Previous studies have suggested young adults’ experiences of homelessness are not gender neutral, with gender identity influencing both health inequities (Aldridge et al., 2018, 2019) and the behaviours in which homelessness persons may engage, or victimisation they may experience (Diette & Ribar, 2018; Heerde & Pallotta‐Chiarolli, 2020; O’Grady & Gaetz, 2009; Watson, 2017). For example, studies involving homeless young women suggest that they are aware of gender performativity in their behaviours (Heerde & Pallotta‐Chiarolli, 2020; Watson, 2017) and actively behave in ways that counter gender norms related to femininity as a means of self‐protection (e.g., carrying a weapon to prevent victimisation or engaging in antisocial behaviours such as physical fights to express strength and power) (Heerde & Pallotta‐Chiarolli, 2020; Watson, 2017). Findings from the Journeys Home project suggests possible gender differences in risk associations between housing insecurity and subsequent violence (Diette & Ribar, 2018).

**FIGURE 1:**
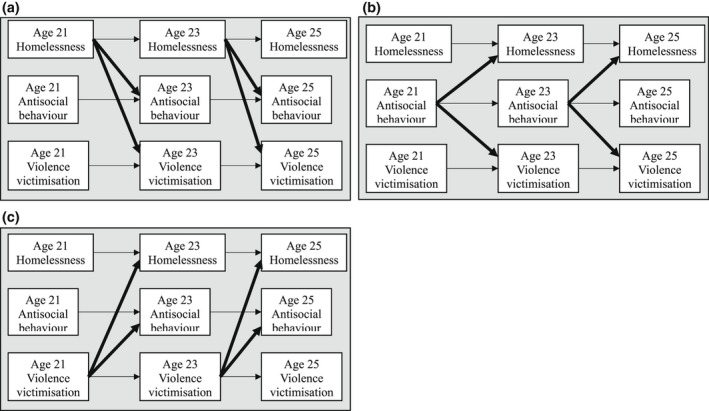
Hypothesised homelessness‐driven (a), antisocial behaviour‐driven (b) and violence victimisation‐driven (c) models. *Note*. Although not shown in the figures for readability, homelessness, antisocial behaviour and violence victimisation are thought to be correlated within age

Knowledge gaps stemming from a lack of longitudinal population‐based studies remain a major challenge and hinder system‐wide capacity to develop targeted, evidenced‐based intervention and prevention approaches that respond to young adult homelessness and its adverse health and social outcomes. In beginning to address these research gaps, we employed cross‐lagged panel models to analyse longitudinal, prospective data from a population‐based sample of young adults at ages 21, 23 and 25 years in Victoria, Australia participating in the International Youth Development Study (IYDS). Three research questions were examined. First, we asked how the prevalence of homelessness changes across young adulthood. Second, we asked about the extent to which homelessness, antisocial behaviour and violence victimisation are associated, both concurrently and across time. We tested three, competing hypotheses: (1) homelessness drives antisocial behaviour and violence victimisation, (2) antisocial behaviour drives homelessness and violence victimisation, and (3) violence victimisation drives homelessness and antisocial behaviour (see Figure 1). Finally, we asked whether associations among homelessness, antisocial behaviour and violence victimisation are similar for males and females.

## 2 METHOD

### 2.1 Participants

This study analysed data drawn from young adults participating in the IYDS, an ongoing longitudinal study examining the development of healthy and problem behaviours among participants from Victoria, Australia, and Washington State, in the United States (US). Only data from the Victorian subsample were included here. Victorian participants have been followed longitudinally into young adulthood.

Full details on the IYDS study design, methods, sampling and recruitment methods have been described elsewhere (McMorris et al., 2007). Briefly, a state‐wide representative sample was achieved using a two‐stage cluster sampling approach in 2002: (1) public and private schools with Grades/Years 5, 7 and 9 (younger, middle and older cohorts, respectively) were randomly selected for recruitment using a probability proportionate to grade‐level size sampling procedure; and (2) randomly selecting one class at the appropriate grade level within each school (McMorris et al., 2007). Across all three cohorts, 3926 eligible Victorian students were approached to participate. Of these, a state‐representative sample of 2884 students (73.5%) consented to and took part in the 2002 survey (51% female).

Data used in this analysis were collected in 2010–11, 2012–13 and 2014–15. Retention rates for each cohort across these study years are presented in Table 1. Participants ranged in age from 18 to 24 years at the 2010–11 survey (*M* = 21.07, SD = 1.67; 54% female), from 19 to 26 years at the 2012–2013 survey (*M* = 23.05, SD = 1.67; 54% female) and 21–28 years at the 2014–15 survey (*M* = 25.00, SD = 1.66; 54% female). For brevity, we refer to these time points as age 21, age 23 and age 25, respectively. Self‐reported sexual orientation was assessed at age 25 only. Across the sample, 81% identified as 100% heterosexual (straight), 13% as mostly heterosexual (straight, but somewhat attracted to people of their own sex), 2% as bisexual (attracted to men and women equally), 1% as mostly homosexual (gay or lesbian, but somewhat attracted to people of the opposite sex), 2% as 100% homosexual (gay or lesbian) and 0.5% as asexual. Most of the Victorian sample identified as Australian (91%).

**TABLE 1:** Retention rates across study cohorts and survey years

	Younger cohort	Middle cohort	Older cohort
2010–11 (age 21)	*n* = 809; 87%	*n* = 826; 84%	*n* = 788; 81%
2012–13 (age 23)	*n* = 787, 85%	*n* = 817, 83%	*n* = 795, 82%
2014–15 (age 25)	*n* = 828, 89%	*n* = 866, 88%%	*n* = 826, 86%

### 2.2 Procedures

Ethics approval was obtained from The University of Melbourne Human Ethics in Research Committee in Australia. Written parental consent and participant assent were obtained at study outset. Participant consent was obtained at each of the young adult surveys. The self‐report young adult survey was completed online and took 50–60 min to complete. The recruitment process included contact and survey visits to correctional facilities and other community‐based services for vulnerable population groups, where some participants resided. Participants were reimbursed $40AUD for their time (at each survey).

### 2.3 Measures

The IYDS survey uses self‐report measures of young adult homelessness, antisocial behaviour and violence victimisation adapted from the Communities That Care youth survey (Arthur et al., 2002; Glaser et al., 2005). Participant demographic data was also collected. The survey was reviewed and adjusted to be developmentally appropriate as the sample aged. The IYDS survey and measures analysed in this study have demonstrated longitudinal validity and reliability with the Victorian young adult sample (Heerde et al., 2020a,b, Toumbourou et al., 2014).

#### 2.3.1 Young adult homelessness

Past year homelessness at ages 21 and 23 years was measured by asking participants, “In the past year, have you ever not had a regular place to live (e.g., homeless)?”. The item “In the past year, have you been homeless (i.e., not had a regular place to live?)” was used to measure homelessness at age 25 years. Response options for each item were dichotomous, “Yes” (1, comparison group) and “No” (0, reference group). Prior studies demonstrate these items accurately reflect the forms of homelessness young adults may experience (e.g., being unsheltered, couch surfing, residing in temporary accommodation (Heerde et al., 2020).

#### 2.3.2 Antisocial behaviour

Six items were used to measure antisocial behaviour at each young adult survey: In the past year (12 months) have you: ‘stolen anything worth more than $5 but less than $50?’; ‘stolen something worth more than $50?’; ‘sold illegal drugs such as marijuana, cocaine, LSD, or heroin?’; ‘attacked someone with the idea of seriously hurting them?’; ‘got into physical fights with other people?’; and ‘beat up someone so badly they probably needed a doctor?’ (Cronbach's *α* = 0.77 at age 21, *α* = 0.75 at age 23 and *α* = 0.74 at age 25). Item response options ranged from “No” (1) through to “Yes, more than once in the past 12 months” (4) and were recoded to reflect “Yes, engaged in antisocial behaviour” (1) and “No, did not engage in antisocial behaviour” (0). Scores for each item were summed to obtain a single scale score at each of age 21, 23 and 25 (0 = No, reference group, 1 = Yes, comparison group).

#### 2.3.3 Violence victimisation

Two items measured violence victimisation at each young adult survey: Have either of these things happened to you in the past year (12 months): ‘been physically attacked’; ‘been threatened with violence’. Response options for both items were dichotomous, “Yes” (1) and “No” (0). Scores for both items were summed to obtain a single scale score at each of age 21, 23 and 25 (0 = No, reference group, 1 = Yes, comparison group). Cronbach's *α* = 0.69 at age 21, *α* = 0.69 at age 23 and *α* = 0.75 at age 25.

#### 2.3.4 Demographic factors

Participants reported their *age* (date of birth) and *sex* (male [0] or female [1]) at each survey. *Study cohort* (younger, middle or older) was established at study outset in 2002. *Family socio*‐*economic status* was created using parent (mother and father) reported level of annual family income (ranging from ‘less than $10,000’ to ‘$200,000 and above’) and highest level of education (e.g., ‘less than secondary school’, ‘completed secondary school’, and ‘completed post‐secondary school’) obtained in phone interviews conducted around the time of the 2002 baseline survey.

### 2.4 Statistical analyses

Stata IC software for Windows version 15.1 (StataCorp LLC, 2017) was used to perform descriptive analyses. Tests of differences in frequencies for homelessness, antisocial behaviour and violence victimisation by sex were conducted using chi‐square analyses. Pooled standard deviations were used to calculate effect sizes for sex differences (Cohen, 1977). Zero‐order correlations were examined to show highly correlated pairs or sets of variables that might result in collinearity in the subsequent analyses. To examine potential attrition bias, associations between attrition and demographic measures and outcomes of interest (homelessness, antisocial behaviour and violence victimisation) were assessed using *t* tests and chi‐square analyses.

A series of prospective, longitudinal cross‐lagged panel models (Selig & Little, 2012) was estimated using Mplus, version 8.2 (Muthén & Muthén, 2017) to examine directional relationships between homelessness, antisocial behaviour and violence victimisation across young adulthood, while controlling for correlations within time‐points and stability (autoregressive effects), across time (Selig & Little, 2012). We used full information maximum likelihood estimation in all analyses to minimise potential bias due to missing data (Muthén & Muthén, 2017). We tested three competing models: (1) a homelessness‐driven model where homelessness predicted later antisocial behaviour and violence victimisation, (2) an antisocial behaviour‐driven model where antisocial behaviour predicted later homelessness and violence victimisation and (3) a violence victimisation‐driven model where violence victimisation predicted later homelessness and antisocial behaviour (Figure 1). Demographic factors were included in each of the analyses, and each model accounted for correlations between observed variables. Model fit indices were examined in accordance with current recommendations (Cangur & Ercan, 2015). The results presented herein are fully standardised.

Finally, we tested whether associations between young adult homelessness, antisocial behaviour and violence victimisation in each of the three cross‐lagged panel models were moderated by sex. We used multiple‐group modelling and chi‐square difference testing in Mplus (Muthén & Muthén, 2017). Differences in model fit between a model constraining parameters to be equal for males and females and an unconstrained model were tested using the *difftest* function.

## 3 RESULTS

### 3.1 Attrition analyses

Participants lost to attrition from age 21 to age 25 were slightly younger at age 21 (20.6 years, *t* = −5.15, *p* < .001) compared to those who were retained (21.1 years). Individuals who reported being homeless at age 21 were significantly less likely to be retained at age 25 (82%, *χ* ^2^ = 4.62, *p* = .032) compared to those who did not report homelessness (88%). There was no significant effect of sex (*χ* ^2^ = 0.33, *p* = .567), childhood family socio‐economic status (*t* = −.37, *p* = .708), age 21 antisocial behaviour (*χ* ^2^ = 1.14, *p* = .285) or age 21 violence victimisation (*χ* ^2^ = 1.45, *p* = .229) on retention from age 21 to age 25.

### 3.2 Tests of difference for homelessness, antisocial behaviour and violence victimisation

Across young adulthood, rates of homelessness were highest at age 21 (6.5%); this rate declined by more than half by age 25 (2.5%; see Table 2). Rates of homelessness did not differ by sex. Rates of antisocial behaviour (39.4%) and violence victimisation (24.2%) were highest at age 21 and declined substantially across ages 23 and 25. Males reported higher rates of antisocial behaviour and violence victimisation than females. Intercorrelations among all study variables were low to moderate, in the expected direction and did not show multicollinearity (*r* ≤ .80; see Appendix A: Table A1). Young adult homelessness, antisocial behaviour and violence victimisation showed moderate to strong cross‐sectional correlations (range *r* = .35–.70).

**TABLE 2:** Summary statistics and tests of sex differences for homelessness, antisocial behaviour and violence victimisation

	Combined sample (*N* = 2423)	Males (*n* = 1109)	Females (*n* = 1314)	*p* Value	*χ* ^2^	Effect size *d*
Past year homelessness (%)						
Age 21	6.50	6.97	6.10	.387	0.749	.04
Age 23	3.92	4.04	3.83	.787	0.073	.01
Age 25	2.47	2.77	2.21	.366	0.818	.04
Antisocial behaviour (%)						
Age 21	39.36	51.82	28.90	<.0001	131.41	.48
Age 23	19.31	29.33	11.05	<.0001	126.24	.48
Age 25	17.35	25.26	10.63	<.0001	92.97	.39
Violence victimisation (%)						
Age 21	24.21	34.61	15.60	<.0001	117.38	.45
Age 23	18.57	27.77	10.98	<.0001	109.66	.44
Age 25	18.54	23.52	14.31	<.0001	35.04	.24

Note

% = percent. *χ* ^2^ = chi‐square. Homelessness, antisocial behaviour and violence victimisation (coded 0 = no, 1 = yes). Sex (coded 0 = male, 1 = female). Statistically significant sex differences were identified using chi‐square tests.

### 3.3 Cross‐lagged panel model results

**FIGURE 2:**
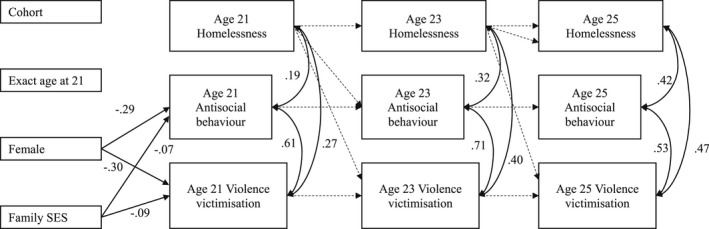
Results from the homelessness‐driven cross‐lagged panel model. *Note*. Dashes indicate non‐significant paths. Solid lines indicate paths significant at *p* < .05. For ease of viewing, non‐significant paths from demographic variables to each of the age 21 variables are not included in the figure, however, they were included in the model

Testing of the three hypothesised models showed that none of the models was supported; results showed no evidence of longitudinal associations among homelessness, antisocial behaviour and violence victimisation. Further, we found no significant autoregressive stability in any of the three variables over time. That is, homelessness, antisocial behaviour and violence victimisation at one time point did not predict homelessness, antisocial behaviour, or violence victimisation, respectively, at subsequent time points. For example, results of the homelessness driven model show a non‐significant path model parameter of −0.04 linking homelessness at ages 23–21 (and −0.08 linking ages 25–23), meaning there was low stability or influence of homelessness from the previous time point (see Appendix B: Table B1). Instead, results for each of the three models showed small to moderate correlations among homelessness, antisocial behaviour and violence victimisation measured concurrently (i.e., cross‐sectionally) at each time point. Figure 2 shows the results of the homelessness‐driven path model. Results from the antisocial behaviour‐ and violence victimisation‐driven models (not shown) yielded an almost identical pattern of significance; significant parameter estimates varied slightly among models at the second or third decimal places. Full results for each of the three models are included in Appendix B: Tables B1–B3. Tests of model equivalence by sex revealed no significant decrement to model fit when parameter estimates were constrained to be equal across sex.

## 4 DISCUSSION

This study is one of few to examine young adult homelessness longitudinally in the context of antisocial behaviour and violence victimisation. The rate of homelessness peaked at the (mean) age 21 survey and declined across the subsequent two time points (mean ages 23 and 25). Cross‐lagged panel modelling showed young adult homelessness, antisocial behaviour, and violence victimisation were not associated longitudinally. Rather, homelessness was associated cross‐sectionally with risk for both antisocial behaviour and violence victimisation. These pathways were not moderated by sex. In sum, results suggest the state of homelessness is associated with temporary vulnerability to harmful and problematic situations involving antisocial behaviour and violence victimisation.

Our findings suggest that the late teens and early 20’s may be a peak period for risk of homelessness, emphasising the need for potential investment in homelessness prevention programs for this age‐group at the population‐level. The transition to adulthood is an important developmental period for establishing relationships and opportunities that set a foundation for healthy adult functioning and achieving adult developmental milestones (e.g., physical and mental health; completing education and entering employment; financial self‐sufficiency, healthy family and social relationships; civic engagement; Scales et al., 2016). Our findings suggest that the experience of homelessness in the early‐mid 20s may be transitory or episodic in this sample. That is, we did not observe continuity in rates of homelessness across time.

Given the low prevalence of homelessness in the current sample and lack of examination of change over time in the prevalence of homelessness in prior studies (Heerde et al., 2021; Shelton et al., 2009; Sznajder‐Murray et al., 2015; Van den Bree et al., 2009; Williams et al., 2019), the current findings should be replicated in future prospective, longitudinal studies using population‐based cohorts. The prevalence of young adult homelessness in Australia remains concerning given its disruptive effects on their health and well‐being (including engagement in antisocial behaviours and experiences of victimisation), meaning that both population‐level prevention efforts (seeking to reduce the number of young adults entering homelessness) and targeted prevention efforts (seeking to reduce the number of young adults established as homeless) are of high importance for measurably reducing population rates of young adult homelessness. Indeed the importance of reducing young adults entry into homelessness, through a focus on prevention and early intervention, has been noted by the Australian Government in their recent response to the *Inquiry into homelessness in Australia* (Australian Government, 2022).

Population‐level prevention efforts linked to social housing policy, accessibility to housing assistance programs and availability of social housing (Gaetz, 2020) as well as Government financial stipends and supplements supporting young adults transitioning from education to employment may lessen the number of young adults entering homelessness (Parliament of the Commonwealth of Australia, 2021). Additionally, targeted approaches such as the provision of safer shelter and accommodation options for those experiencing homelessness are likely to mitigate the physical risks of homelessness, for example, the risk of being a victim of violence. Identifying those young adults that are at especially high risk for both homelessness and homelessness‐related antisocial behaviour and victimisation is important in the development of flexible and strategic health and social policies and programs that can be scaled and varied proportionate to needs.

Our findings illustrate the potential of using longitudinal, population‐level data to examine not only the timing of homelessness but also its relationship to antisocial behaviour and violence victimisation. In this study, homelessness appears to be acutely (cross‐sectionally) associated with increased risk for violence victimisation and for antisocial behaviours. Homeless young adults report considerable vulnerability to violence victimisation and engaging in a range of survival‐related antisocial behaviours (such as theft of food or money to meet survival needs), or violence in response to threats, vulnerability or marginalisation (Gaetz, 2004, Heerde & Pallotta‐Chiarolli, 2020, Kipke et al., 1997; McCarthy & Hagan, 1991; Tyler et al., 2018). Indeed, the items we have used to measure antisocial behaviour (e.g., stealing) and violence victimisation (e.g., being threatened) in the current study, reflect survival‐related behaviours and threats of harm that have been reported as being associated with homelessness in prior studies with selective samples (e.g., Baron, 2003; Kipke et al., 1997, Heerde & Pallotta‐Chiarolli, 2020, Tyler et al., 2018).

It is also possible that these situational triggers for survival‐related antisocial behaviours (e.g. threats to self) are more common when these behaviours are socially approved and accepted as normal in the social context of homelessness when they occur (e.g., by homeless peers; Heerde & Pallotta‐Chiarolli, 2020). Interdependent relationships with homeless peers may provide important sources of emotional support and physical protection, but also may have considerable influence on antisocial behaviour and violence victimisation, either through normalisation of such behaviour or because peers themselves may be a source of violence exposure for homeless young adults (Heerde & Pallotta‐Chiarolli, 2020; Kipke et al., 1997). Additional research clarifying the role of peers is needed.

One reason for the lack of longitudinal associations between homelessness, survival‐related antisocial behaviour, and violence victimisation may lie in confounding variables characterising background social context. Homelessness prevention and intervention requires an ecological approach (Heerde et al., 2021). The current findings support prior research demonstrating that antecedents of stable antisocial behaviour occur through neurodevelopmental stressors in childhood and adolescence (Fairchild et al., 2013). It is also possible that the effects of broader social‐ecological antecedents of homelessness experienced in adolescence subsequently influence survival‐related antisocial behaviour and violence victimisation in young adulthood (e.g., educational risk, unemployment, peer group affiliations; Heerde et al., 2021, 2022). Where antecedents such as these are identified, they can and should be targeted by preventive intervention at this stage of development (i.e., adolescence). Other homelessness‐related confounders, for example, current substance use and/or mental health problems, may also influence antisocial behaviour and violence victimisation (Heerde & Hemphill, 2015). While young adult homelessness represents an immediate situational vulnerability to antisocial behaviour and victimisation, it does not appear to change developmental trajectories.

The field of prevention science has demonstrated that prevention is “never too early, never too late.” (Loeber & Farrington, 1998). Thus, adolescence is not too early to implement homelessness prevention, nor is intervention at later stages of development, such as young adulthood, too late. An important direction for future studies examining longitudinal associations between homelessness, survival‐related antisocial behaviour and violence victimisation should seek to confirm the current findings, investigate possible homelessness‐related confounders, and include detailed information on potential adolescent social‐ecological antecedents. Investigations of this nature will provide a stronger basis on which to examine potential mechanisms that may explain young adult pathways to homelessness and the relationship between homelessness, survival‐related antisocial behaviour and violence victimisation across time. The findings arising from such analyses will also inform intervention timing and targets critical to the development, testing and implementation of prevention programming aimed at reducing rates of homelessness, antisocial behaviour and violence victimisation.

### 4.1 Study strengths and limitations

The longitudinal study design and analysis of repeated measures has enabled study of the change in the rates of homelessness across young adulthood, and the examination of longitudinal directional associations between homelessness, antisocial behaviour and violence victimisation. Data were collected from a population‐based cohort, which was representative of Grade/Year 5–9 students in Victoria at the time of study commencement and has high retention rates. The IYDS survey has demonstrated longitudinal validity in the Victorian sample (Heerde et al., 2020a, 2021, Hemphill et al., 2011; Toumbourou et al., 2014). The use of cross‐lagged panel models permitted the investigation of how homelessness, antisocial behaviour and violence victimisation are concurrently and longitudinally related to each other, enhancing the specificity of implications for intervention and prevention targets.

We used a single, dichotomous item to measure young adult homelessness; although measures such as this are common in homelessness research it has limited our capacity to analyse how different forms of homelessness may be associated with antisocial behaviour or violence victimisation. Prior studies have demonstrated the homelessness item used here, accurately reflects the types of homelessness experienced by young adults (e.g. couch surfing, residing in temporary accommodation, rough sleeping; Heerde et al., 2020). The rate of young adult homelessness (approximately 6% of the sample at age 21) is lower than the national average (Australian Institute of Health & Welfare, 2020) and is likely to be an underestimation (Hall et al., 2020). This study did not examine associations of chronic homelessness with antisocial behaviour or victimisation, which may differ from those observed here. Participants experiencing homelessness are likely to be a high‐risk group for attrition and were significantly less likely to be retained here. However, retention among individuals reporting homelessness was quite high (82%), and the difference in retention rates between individuals reporting and not reporting homelessness (6%) was small. Thus, we do not think that the observed lack of continuity in homelessness over time is explained by attrition. Our analyses may have been underpowered to detect small effects, including moderation by sex, as significant. Nonetheless, we were able to detect significant path model parameters as small as 0.07 (linking family socioeconomic status and age 21 antisocial behaviour).

Prior research has noted gendered experiences of homelessness are associated with survival‐related antisocial behaviours and victimisation experiences (Diette & Ribar, 2018; O’Grady & Gaetz, 2009; Watson, 2017). The results suggest that these relationships did not differ by gender identity in our sample; however, we did find that those who identified as female were less likely to engage in antisocial behaviour or be victimized at age 21. This may suggest that young adult females in our sample began with lower risk compared to males.

Owing to the high proportions of young adults identifying as being Australian and identifying as heterosexual (>90%), we intentionally did not examine differences in associations by ethnicity or sexual identity. Subdividing the low number of young adults reporting homelessness would have yielded small cell sizes and negatively affected power to detect differences by ethnicity or sexual identity, limiting the reliability of findings. The analyses conducted here should be replicated in future longitudinal studies using population‐level samples and include a detailed examination of the potential role of gender, ethnic and sexual identities.

Our analyses focussed exclusively on testing the hypothesised relationships between young adult homelessness, antisocial behaviour and violence victimisation. Mapping longitudinal pathways to young adult homelessness and testing the role of antecedents that increase or moderate risk for young adult homelessness (e.g., childhood trauma, out‐of‐home care placement) were beyond the scope of the current study. Likewise, we have not sought to estimate the impact of young adult homelessness on later health or social outcomes (e.g., drug and alcohol addiction, mental ill‐health, incarceration). The use of prospective, longitudinal population‐based data to map developmental pathways into homelessness and estimate the causal impact of homelessness on later outcomes and functioning requires detailed study. The measures analysed in this study are based on self‐report data; however, this is considered reliable in studies of young adults and for the measures analysed here (Jolliffe et al., 2003). The study findings are generalizable only to the state and sample analysed.

### 4.2 Study implications

Rates of homelessness in Australian young adults continue to be of concern. The current study informs the developmental pattern of homelessness, revealing the highest young adult prevalence at age 21, and the transitory or episodic nature of homelessness during young adulthood. The consistent cross‐sectional associations with antisocial behaviour and victimisation through to the mid‐20s reinforces evidence that the state of homelessness increases vulnerability to risk for antisocial behaviour and violence victimisation and risk for related physical and psychological harm that is likely to extend well into adulthood (Aldridge et al., 2018; Heerde et al., 2020b). That is, our findings suggest that homelessness in young adulthood is associated with important concurrent behavioural and physical risks that increase the likelihood of negative and potentially long‐term complications, such as poor health outcomes (e.g., physical injury, mental ill‐health) and involvement with the justice system.

Our findings point to the need for investment in both *population*‐*level* and *targeted* homelessness prevention and intervention strategies. In prior studies analysing data from participants in the IYDS, we have successfully identified malleable life‐course antecedents of homelessness across multiple‐interacting social‐ecological contexts. Findings from multivariable regression and path modelling showed academic failure and school suspension, peer substance use and low attachment to one's neighbourhood in adolescence predicted young adult homelessness (Heerde et al., 2021, 2022). The implementation of tested‐effective frameworks and programs (Blueprints for Healthy Youth Development, 2019) known to reduce these predictors is important to informing evidenced‐based prevention and intervention approaches seeking to reduce young adult homelessness, and subsequently antisocial behaviour and violence victimisation among homeless young adults.

The current results suggest that the timing of prevention and intervention strategies is important for both reducing entry into homelessness and to decreasing the amount of time young adults’ experience homelessness. Targeted prevention and intervention strategies that seek to provide point in time assistance and support homeless young adults are critical to minimising the point‐in‐time association of homelessness with both antisocial behaviour and violence victimisation and to minimising the impact of antisocial behaviour and violence victimisation (e.g., financial assistance; safe shelter). These strategies should acknowledge antisocial behaviours and violence victimisation may be associated with social expectations and rules within homeless communities that see these being accepted as normal when they occur. Findings resulting from the analysis of longitudinal, prospective population‐level data will allow for a stronger evidence base that can be used to more efficiently allocate resources to areas likely to have the biggest impact on reducing young adult homelessness and increase support to those exposed to violent victimisation and survival‐related antisocial behaviour.

## AUTHOR CONTRIBUTIONS

**Jess Heerde:** Conceptualization; Formal analysis; Funding acquisition; Investigation; Writing – original draft; Writing – review & editing. **Jennifer A Bailey:** Conceptualization; Formal analysis; Funding acquisition; Investigation; Project administration; Writing – review & editing. **John Toumbourou:** Funding acquisition; Project administration; Writing – review & editing. **George C Patton:** Writing – review & editing.

## APPENDIX A

**TABLE A1** Zero‐order associations among study variables

**Table:**

	1	2	3	4	5	6	7	8	9	10	11	12	13
1. Study cohort	–	**0.96**	−0.001	0.03	0.01	0.01	<−0.001	**0.06**	**0.09**	0.04	−0.01	**0.10**	**0.05**
2. Age		–	−0.04	−0.02	0.02	0.02	0.004	**0.07**	**0.09**	0.04	0.001	**0.09**	**0.04**
3. Sex			–	−0.04	−0.04	−0.08	0.07	**−0.36**	0.003	0.05	**−0.37**	−0.04	−0.03
4. Family SES				–	**−0.04**	0.01	−0.01	**−0.04**	−0.01	−0.02	**−0.06**	0.01	.002
5. Age 21 homelessness					–	−0.02	**−0.22**	**0.19**	0.03	0.01	**0.28**	0.04	−0.10
6. Age 23 homelessness						–	−0.01	−0.002	**0.35**	0.05	0.01	**0.37**	0.09
7. Age 25 homelessness							–	−0.14	−0.02	**0.43**	−0.01	−0.16	**0.45**
8. Age 21 antisocial behaviour								–	0.01	−0.05	**0.65**	0.07	−0.01
9. Age 23 antisocial behaviour									–	0.05	0.01	**0.70**	−0.07
10. Age 25 Antisocial behaviour										–	−0.04	**−0.003**	**0.53**
11. Age 21 violence victimisation											–	0.03	−0.07
12. Age 23 violence victimisation												–	−0.003

Note

Statistically significant associations in bold (at least *p* < .05). Homelessness, antisocial behaviour and violence victimisation (coded 0 = no, 1 = yes). Sex (coded 0 = male, 1 = female). Point biserial correlations were performed between a dichotomous variable and a continuous variable. Tetrachoric correlations were performed between two dichotomous variables. Pearson correlations were performed between two continuous variables.

## APPENDIX B

**TABLE B1** Parameter estimates from the homelessness‐driven cross‐lagged panel model

**Table:**

Association estimated	Standardized estimate	SE	*p*‐Value
Age 21 homelessness predicted by			
Study cohort	−0.211	0.138	.127
Female (referent: male)	−0.020	0.041	.627
Age (years)	0.254	0.142	.074
Family socioeconomic status	−0.081	0.041	.050
Age 21 antisocial behaviour predicted by			
Study cohort	−0.045	0.094	.632
Female (referent: male)	−0.289	0.024	<.0001
Age (years)	0.121	0.094	.197
Family socioeconomic status	−0.067	0.026	.009
Age 21 victimisation predicted by			
Study cohort	−0.088	0.101	.381
Female (referent: male)	−0.296	0.026	<.0001
Age (years)	0.076	0.100	.450
Family socioeconomic status	−0.092	0.028	.001
Age 23 homelessness predicted by Age 21			
Homelessness	−0.038	0.090	.673
Age 23 antisocial behaviour predicted by Age 21			
Homelessness	0.037	0.063	.557
Antisocial behaviour	−0.009	0.044	.883
Age 23 violence victimisation predicted by Age 21			
Homelessness	−0.018	0.067	.790
Violence victimisation	0.053	0.048	.268
Age 25 homelessness predicted by Age 23			
Homelessness	−0.075	0.120	.531
Age 25 antisocial behaviour predicted by Age 23			
Homelessness	0.007	0.085	.932
Antisocial behaviour	0.044	0.059	.464
Age 25 violence victimisation predicted by Age 23			
Homelessness	0.120	0.090	.180
Violence victimisation	−0.097	0.067	.147
Correlations specified in the model			
Age 21 homelessness with Age 21			
Antisocial behaviour	0.185	0.051	<.0001
Violence victimisation	0.270	0.051	<.0001
Age 21 antisocial behaviour with Age 21			
Violence victimisation	0.611	0.027	<.0001
Age 23 homelessness with Age 23			
Antisocial behaviour	0.315	0.064	<.0001
Violence victimisation	0.404	0.061	<.0001
Age 23 antisocial behaviour with Age 23			
Violence victimisation	0.714	0.027	<.0001
Age 25 homelessness with Age 25			
Antisocial behaviour	0.415	0.068	<.0001
Violence victimisation	0.473	0.069	<.0001
Age 25 antisocial behaviour with Age 25			
Violence victimisation	0.534	0.037	<.0001

Note

Model fit statistics: *χ* ^2^ (41, *N* = 2328) = 41.83, *p* = .4346, Comparative Fit Index = 0.999, Tucker‐Lewis Index = 0.999, Root‐Mean‐Square Error of Approximation = 0.003. Correlations among exogenous demographic characteristics are not estimated in the model; the observed correlations between these variables are taken into account by Mplus. SE = standard error. Homelessness, antisocial behaviour and violence victimisation (coded 0 = no, 1 = yes). Female (coded 0 = male, 1 = female).

**TABLE B2** Parameter estimates from the antisocial behaviour‐driven cross‐lagged panel model

**Table:**

Association estimated	Standardized estimate	SE	*p*‐value
Age 21 homelessness predicted by			
Study cohort	−0.213	0.139	.125
Female (referent: male)^	−0.022	0.041	.591
Age (years)^	0.252	0.142	.077
Family socioeconomic status^	−0.079	0.041	.058
Age 21 antisocial behaviour predicted by			
Study cohort	−0.026	0.094	.786
Female (referent: male)^	−0.290	0.024	<.0001
Age (years)^	0.113	0.094	.230
Family socioeconomic status^	−0.068	0.026	.008
Age 21 violence victimisation predicted by			
Study cohort	−0.110	0.101	.277
Female (referent: male)^	−0.295	0.026	<.0001
Age (years)^	0.086	0.100	.394
Family socioeconomic status^	−0.092	0.028	.001
Age 23 homelessness predicted by Age 21			
Homelessness	−0.019	0.097	.843
Antisocial behaviour	0.005	0.073	.948
Age 23 antisocial behaviour predicted by Age 21			
Antisocial behaviour	0.016	0.042	.695
Age 23 violence victimisation predicted by Age 21			
Antisocial behaviour	0.095	0.067	.156
Violence victimisation	−0.058	0.069	.403
Age 25 homelessness predicted by Age 23			
Antisocial behaviour	−0.098	0.110	.375
Homelessness	0.019	0.158	.906
Age 25 antisocial behaviour predicted by Age 23			
Antisocial behaviour	0.046	0.051	.368
Age 25 violence victimisation predicted by Age 23			
Antisocial behaviour	−0.198	0.106	.061
Violence victimisation	0.151	0.104	.146
Correlations specified in the model			
Age 21 homelessness with Age 21			
Antisocial behaviour	0.186	0.051	<.0001
Violence victimisation	0.267	0.051	<.0001
Age 21 antisocial behaviour with Age 21			
Violence victimisation	0.611	0.027	<.0001
Age 23 homelessness with Age 23			
Antisocial behaviour	0.313	0.066	<.0001
Violence victimisation	0.400	0.060	<.0001
Age 23 antisocial behaviour with Age 23			
Violence victimisation	0.713	0.027	<.0001
Age 25 homelessness with Age 25			
Antisocial behaviour	0.419	0.067	<.0001
Violence victimisation	0.460	0.063	<.0001
Age 25 antisocial behaviour with Age 25			
Violence victimisation	0.539	0.037	<.0001

Notes

Model fit indices: *χ* ^2^ (41, *N* = 2328) = 40.18, *p* = .5067, Comparative Fit Index = 1.000, Tucker‐Lewis Index = 1.001, Root‐Mean‐Square Error of Approximation = <0.0001. Correlations among exogenous demographic characteristics are not estimated in the model; the observed correlations between these variables are taken into account by Mplus. SE = standard error. Homelessness, antisocial behaviour and violence victimisation (coded 0 = no, 1 = yes). Female (coded 0 = male, 1 = female).

^ = measured at Grade/Year 7.

**TABLE B3** Parameter estimates from the violence victimisation‐driven cross‐lagged panel model

**Table:**

Association estimated	Standardized estimate	SE	*p*‐Value
Age 21 homelessness predicted by			
Study cohort	−0.213	0.139	.124
Female (referent: male)^	−0.022	0.041	.598
Age (years)^	0.252	0.142	.076
Family socioeconomic status^	−0.078	0.041	.058
Age 21 antisocial behaviour predicted by			
Study cohort	−0.049	0.094	.601
Female (referent: male)^	−0.290	0.024	<.0001
Age (years)^	0.122	0.094	.196
Family socioeconomic status^	−0.066	0.026	.010
Age 21 violence victimisation predicted by			
Study cohort	−0.082	0.101	.419
Female (referent: male)^	−0.296	0.026	<.0001
Age (years)^	0.074	0.100	.458
Family socioeconomic status^	−0.093	0.028	.001
Age 23 homelessness predicted by Age 21			
Homelessness	−0.031	0.104	.764
Violence victimisation	0.012	0.080	.886
Age 23 antisocial behaviour predicted by Age 21			
Antisocial behaviour	−0.041	0.066	.534
Violence victimisation	0.045	0.069	.518
Age 23 violence victimisation predicted by Age 21			
Violence victimisation	0.050	0.043	.240
Age 25 homelessness predicted by Age 23			
Violence victimisation	−0.145	0.113	.202
Homelessness	0.077	0.159	.627
Age 25 antisocial behaviour predicted by Age 23			
Violence victimisation	−0.081	0.104	.435
Antisocial behaviour	0.124	0.103	.229
Age 25 violence victimisation predicted by Age 23			
Violence victimisation	−0.036	0.050	.464
Correlations specified in the model			
Age 21 homelessness with Age 21			
Antisocial behaviour	0.185	0.051	<.0001
Violence victimisation	0.269	0.051	<.0001
Age 21 antisocial behaviour with Age 21			
Violence victimisation	0.611	0.027	<.0001
Age 23 homelessness with Age 23			
Antisocial behaviour	0.325	0.064	<.0001
Violence victimisation	0.390	0.062	<.0001
Age 23 antisocial behaviour with Age 23			
Violence victimisation	0.712	0.027	<.0001
Age 25 homelessness with Age 25			
Antisocial behaviour	0.418	0.067	<.0001
Violence victimisation	0.463	0.063	<.0001
Age 25 antisocial behaviour with Age 25			
Violence victimisation	0.532	0.036	<.0001

Notes

Model fit indices: *χ* ^2^ (41, *N* = 2328) = 41.36, *p* = .4550, Comparative Fit Index = 1.000, Tucker‐Lewis Index = 1.000, Root‐Mean‐Square Error of Approximation = 0.002. Correlations among exogenous demographic characteristics are not estimated in the model; the observed correlations between these variables are taken into account by Mplus. SE = standard error. Homelessness, antisocial behaviour and violence victimisation (coded 0 = no, 1 = yes). Female (coded 0 = male, 1 = female).

^ = measured at Grade/Year 7.
